# Evaluation of the Approach to Rectal Injuries During Pelvic Uro-Oncological Surgery

**DOI:** 10.3390/jcm15031080

**Published:** 2026-01-29

**Authors:** Ata Özen, Süleyman Öner, Necdet Fatih Yaşar, Utku Bekyürek, İyimser Üre

**Affiliations:** 1 Department of Urology, Faculty of Medicine, Eskisehir Osmangazi University, Eskisehir 26040, Turkey; dr.suleymanoner@gmail.com (S.Ö.); ubekyurek@gmail.com (U.B.); iyimserure@gmail.com (İ.Ü.); 2 Department of General Surgery, Faculty of Medicine, Eskisehir Osmangazi University, Eskisehir 26040, Turkey; nfyasar@gmail.com

**Keywords:** rectal injury, pelvic uro-oncological surgery, radical prostatectomy, radical cystectomy

## Abstract

**Background/Objectives**: Rectal injuries are rare but serious complications during pelvic surgeries. This study aimed to share our experience with primary repair of rectal injuries occurring during pelvic uro-oncological surgeries. **Methods**: A total of 494 patients who presented to our clinic with diagnoses of non-metastatic bladder tumors and prostate cancer and underwent radical prostatectomy, and 214 patients who underwent radical cystectomy between 2010 and 2024 were included in the study. Data from 15 patients who experienced rectal injury during pelvic uro-oncological surgery were retrospectively reviewed. Patients scheduled for radical prostatectomy received rectal enemas twice a day (morning and evening) the day before surgery. Patients scheduled for radical cystectomy received a liquid diet for three days preoperatively, along with rectal enemas morning and evening. **Results**: Rectal injury occurred in 7 (1.4%) patients during radical prostatectomy and in 8 (3.7%) patients during radical cystectomy. All rectal injuries were detected intraoperatively. The defect was closed in two layers: muscular and serosal. Patients had no active complaints during follow-up, and no rectourinary fistula was observed. **Conclusions**: Intraoperative detection of rectal injury during pelvic uro-oncological surgery and high-quality primary repair of the defect play a critical role in preventing morbidity and mortality associated with rectal injury. Preoperative mechanical bowel preparation reduces contamination of the procedure, improves the visibility of the defect, and allows for high-quality primary repair.

## 1. Introduction

Rectal injuries (RIs) are rare but serious complications that can occur during pelvic surgeries. Urological, gynecological, and general surgical procedures are the most common causes of iatrogenic bowel injury [1]. A portion of iatrogenic RIs resulting from pelvic surgeries is caused by urological procedures. Due to the close anatomical relationship between the rectum, bladder, and prostate, there is a risk of injury, particularly during radical prostatectomy (RP) and radical cystectomy (RC). RIs may occur if the tumor extends beyond the organ and invades the rectum, or during RP and RC if the Denonvilliers’ fascia is not properly incised, especially during dissection of the prostatic base or apex [2,3]. Intraoperative detection of RIs is extremely important; once confirmed, immediate repair of the defect is recommended [2]. Undetected RI can significantly increase morbidity and even mortality through wound infections, pelvic abscess, fistula, and sepsis [4].

During pelvic surgery, the diagnosis of RI is made by visualizing a defect in the rectal wall or the presence of fecal content. In addition, intraoperative endoscopy or air leak testing can also be performed [1]. Regardless of the surgical technique, it is crucial that RI is recognized and managed as early as possible. In most case series in the literature, it has been reported that the rectal defect was primarily repaired intraoperatively and that colostomy was not required [1,2,5]. Colostomy should not be considered the standard approach for all patients with intraoperatively detected RI. It should be considered in cases of large rectal defects (>2 cm), a history of pelvic radiotherapy, prior BPH surgery, or the presence of rectal infiltration. According to the literature, colostomy was preferred in the treatment of only about 10% of patients with RI [6].

The aim of this study is to share our experience with primary repair in RI occurring during pelvic uro-oncological (PUOS) surgeries.

## 2. Materials and Methods

A total of 708 patients who presented to our clinic between 2010 and 2024 with diagnoses of non-metastatic bladder cancer and prostate cancer and underwent PUOS (open RC, retropubic RP) were included in the study. The data of 15 patients who developed RI during PUOS were retrospectively analyzed. All surgical operations were performed by the same surgeon.

For patients planned for RP, rectal enemas were performed twice, in the morning and evening, one day prior to surgery, and routine surgical antibiotic prophylaxis (Cefazolin) was administered. For patients planned for RC, a liquid diet for three days preoperatively, along with morning and evening rectal enemas, was given, and antibiotic prophylaxis (Cefuroxime + Metronidazole) was applied. At the beginning of both operations, a rectal tube was placed 6–7 cm inside the anal margin of the patients. Postoperatively, all cases underwent direct observation for RI control, and in suspicious cases, the surgical area was filled with saline solution, and air was introduced via a 50 cc syringe from the rectal tube (air test) to check for rectal integrity. In cases where RI was detected, rectal defect repair was performed after prostate removal, before vesicourethral anastomosis, or after cystectomy. The defect was closed with primary 2/0 polyglactin absorbable sutures in two layers, consisting of muscular and serosal layers. In cases of RI occurring during both operations, inflammation was monitored with blood tests, and antibiotic treatment with cefuroxime and metronidazole was administered for 7–10 days in the postoperative period. On the third postoperative day, patients without active complaints and with no pathological findings during abdominal examination were given small amounts of water, and those who tolerated it were started on Regimen 1 (water only). In follow-up visits, patients without issues were progressively given Regimen 2 (water and liquid foods like soup) and then Regimen 3 (all food and drinks). Patients who tolerated Regimen 3, had gas and stool passage, and showed no active complaints during follow-up were discharged with recommendations. The catheters of patients who underwent RP were removed on the 12th postoperative day following cystogram control.

Patients who underwent RP with and without RI were compared in terms of age, preoperative prostate-specific antigen (PSA) levels, RP International Society of Urological Pathology (ISUP) grade, presence of metastatic lymph nodes, and RP pT stage. Similarly, patients who underwent RC with and without RI were compared in terms of age, gender, initial transurethral resection of bladder tumors (TUR-BT) stage, initial TUR-BT variant histology, neoadjuvant chemotherapy, presence of metastatic lymph nodes, concomitant prostate cancer, and pT stage.

The patients who developed RI during RP were retrospectively analyzed in terms of their age, body mass index, comorbidities, digital rectal examination findings, preoperative total PSA levels, the time between biopsy and surgery, the size of the RI, the stage of the surgery at which the injury occurred, the duration of surgery, the length of hospital stay, specimen pathology reports and stages, and European Association of Urology (EAU) risk classifications.

The patients who developed RI during RC were retrospectively analyzed in terms of their age, body mass index, comorbid systemic diseases, initial TUR-BT localization and pathology, size of the RI, the stage of the surgery at which the injury occurred, duration of surgery, length of hospital stay, early postoperative complications, and specimen pathology reports.

All statistical analyses were performed using IBM SPSS Statistics v23 software for Windows (SPSS Inc., Chicago, IL, USA). Categorical variables were expressed as patient count (frequency) and percentage (%). Descriptive statistics for continuous numerical variables were presented as mean ± standard deviation. Statistical differences between groups were analyzed using the Student’s *t*-test. Evaluation of multi-cell cross tables was performed using the Chi-square test, and results were considered statistically significant when the *p*-value was <0.05.

## 3. Results

Out of the 708 patients who underwent PUOS, 494 underwent retropubic RP and 214 underwent open RC. During these surgeries, iatrogenic RI was observed in a total of 15 patients (2.1%). RI occurred in 7 patients (1.4%) during RP and in 8 patients (3.7%) during RC. All cases of RI were detected intraoperatively. Preoperative evaluation revealed rectal invasion in 4 patients, and controlled rectal incision was performed for tumor resection to ensure negative surgical margins. None of the patients had a history of radiotherapy or pelvic surgery.

When comparing patients who underwent RP with and without RI in terms of age, preoperative PSA levels, RP ISUP grade, presence of metastatic lymph nodes, and RP pT stage, no significant differences were observed. Similarly, when comparing patients who underwent RC with and without rectal injury in terms of age, gender, initial TUR-BT stage, initial TUR-BT variant histology, neoadjuvant chemotherapy, presence of metastatic lymph nodes, concomitant prostate cancer, and pT stage, no significant differences were observed (Table 1).

The average age of patients in the iatrogenic RI group during RP was 63 (±5.97). The average body mass index (BMI) of the patients was 26.7 (±1.98). Two patients had a diagnosis of essential hypertension, and no other comorbidities were present in the remaining patients. Digital rectal examination revealed hardness at the prostatic base in 5 patients. The average total PSA level of the patients was 21.2 (±18.6) ng/mL. The average time between prostate biopsy and RP was 4.5 months (±3.69). The average size of the RI was 2 cm (±0.9). RI occurred during prostate base dissection in 71% of the cases, and during apex dissection in 29% of the cases. The average case duration was 192.4 (±7.56) minutes. Identifying and repairing the RI affected the surgery time by approximately 20–25 min. According to pathology reports, 4 patients (57%) had tumor extension at the surgical margin, and 2 patients (28%) had metastatic lymph nodes. The average volume of the excised prostate specimens was 67.9 (±23.6). Three patients (23%) were classified as high risk according to the EAU risk classification. The average hospital stay for the patients was 12 (±2.52) days. The average follow-up period in outpatient clinics was 81.86 (±54.7) months. No local recurrence was observed in the follow-up of patients who were thought to have rectal tumor invasion before surgery. None of the patients had active complaints during follow-up, and no recto-urinary fistulas (RUF) were observed (Table 2).

The average age of patients who developed RI during RC was 65.7 (±8.53). The average BMI of the patients was 24.75 (±3.15). Regarding comorbidities, two patients had a history of coronary artery disease and hypertension, and one patient had acute lymphoblastic leukemia. Only one patient (12.5%) underwent RC due to non-muscle invasive bladder cancer, unresponsive to intravesical immunotherapy, while the other seven patients (87.5%) underwent RC due to muscle-invasive bladder cancer. The average size of the RI was 2 (±0.76) cm. RI occurred during posterior dissection in 75% of the cases. The average operation duration was 270.6 (±25.69) minutes. Identifying and repairing the RI affected the surgery time by approximately 20–25 min. Postoperatively, 2 patients (25%) had wound site infections (Clavien–Dindo grade IIIa), 1 patient (12.5%) developed sepsis (grade IV), and 1 patient (12.5%) had gastrointestinal bleeding (grade I). The pathological stage of 3 patients (37.5%) was reported as T0, 2 patients (25%) had a pathological stage of T4a, and 3 patients (37.5%) were found to have variant cytology. All patients had negative surgical margins. Two patients (25%) had metastatic lymph nodes. The average hospital stay for the patients was 22.5 (±4.14) days. The average follow-up period in outpatient clinics was 55.5 (±25.66) months. No local recurrence was observed in the follow-up of patients who were thought to have rectal tumor invasion before surgery. None of the patients had active complaints during follow-up, and no RUFs were observed (Table 3).

## 4. Discussion

The anatomical location of the prostate poses a risk for RI during prostatectomy. RI is a rare but potentially devastating complication following RP. The incidence of RI ranges from 0% to 20.39%, with significant variations between studies [6]. A study conducted by Barashi et al. demonstrated that the risk of RI during RP is significantly lower in high-volume centers (those performing >43 cases annually) compared to low-volume centers (those performing <43 cases annually) [7]. In our study, RI was detected in 7 (1.4%) out of 494 RP cases. Although the incidence of RI in our study is relatively low, it was found to be consistent with the literature. When reviewing recent years, we have been performing over 60 cases annually, which may explain the relatively low incidence of RI.

It has been emphasized that RIs occurring during laparoscopic cystectomy are particularly likely during the dissection of the prostate apex from the rectum [3]. In a meta-analysis compiling complications of RC, injury to adjacent organs was observed in 1% of cases [8]. In our study, RI was detected in 8 (3.7%) patients during RC. With an increase in the number of cases and the experience gained over time, we expect this rate to decrease.

It is believed that several risk factors influence the development of RI. Most of these risk factors are related to the experience of the surgeon in performing RP. Risk factors for RI include previous pelvic surgeries (rectal or prostate surgery) or radiation therapy, locally advanced tumors, especially high-risk prostate cancer, the surgeon’s expertise and learning curve, salvage surgery, patient characteristics (obesity and advanced age), the duration between prostate biopsy and RP, hospital volume, and surgical volume [2]. In our study, when we examined the patients who underwent RP and RC for potential risk factors of RI, we were unable to draw any significant conclusions. This result may be due to the substantial difference in the number of patients with and without RI. However, the difference may not have reached a statistically significant difference due to the low number of cases in which rectal injury was detected. Therefore, multivariate analysis could not be performed.

The management and prognosis of RI occurring during RP significantly differ based on the timing of detection (intraoperative vs. postoperative). Intraoperative detection of RI is associated with a significant reduction in the risk of severe postoperative complications and the subsequent development of RUF [9]. Therefore, all measures aimed at detecting RI intraoperatively should be strongly considered and implemented. A catheter can be placed into the rectum at the beginning of the surgical procedure to assist the surgeon in identifying rectal mucosal perforation. If RI is suspected or there is a visible thin rectal wall, an air test should always be performed. Prior to this, the surgical field should be irrigated and filled with normal saline solution to remove pelvic blood clots and identify actively bleeding vessels. Air can be insufflated into the rectum using a rectal catheter. The detection of air bubbles escaping from the rectum clearly indicates the presence of a small rectal lesion [10]. Before PUOS, we routinely placed a 20 Fr rectal tube into the rectum and checked the integrity of the rectum through direct visualization postoperatively. In cases suspicious for RI, we performed an air test. Due to these measures, we detected all cases of RI intraoperatively and performed primary repair during the surgery. In the long-term follow-up of the patients, we did not encounter any serious complications, such as RUF.

In cases of RI detected during RP, the lesion size ranges from 1 to 3 cm in the open series by Topaktas et al., while in the robotic series by Khetepral et al., it ranges from 0.3 to 2 cm [11,12]. In our study, the average sizes of RI occurring during RP and RC were measured as 2 (±0.9) cm and 2 (±0.76) cm, respectively, and were found to be consistent with the literature.

No specific antibiotic prophylaxis has been indicated for patients at risk of RI during RP. To reduce the severity of RI by subsequently lowering the risk of infectious complications and/or delayed colostomy, some authors have recommended mechanical bowel preparation [12,13]. In our practice, we routinely perform mechanical bowel preparation for all patients prior to PUOS. This reduces the risk of contamination during the procedure and allows for better visualization of any potential rectal defects during PUOS. As a result, primary repair of the rectal defect can be performed more controlled and with higher quality.

The treatment of RI depends on the timing of diagnosis, the size of the defect, and the patient’s clinical condition. There are no randomized prospective studies or algorithms to guide decision-making; however, according to the latest review by Leevan et al., studies comparing primary repair and colostomy have not reported worse outcomes for primary repair. As a general rule from trauma literature, if an iatrogenic injury is small and recognized intraoperatively, primary repair of the defect is generally considered appropriate [1]. Kheterpal et al. reported 10 cases of RI from over 4400 RP and managed all cases of RI with intraoperative detection and management [12]. During RP, RI can occur at the beginning of the procedure during seminal vesicle dissection or during the dissection of the posterior plane between the prostate and the rectal wall. In this case, some authors have suggested repairing the lesion at this stage before further mobilization of the prostate [14]. Some authors, however, have recommended repairing the defect after the prostate has been removed [11]. Regardless of the approach used, the rectal defect should be closed before completing the vesicourethral anastomosis [9]. The surgical area should be thoroughly irrigated with a large amount of saline solution or povidone-iodine [5,11,12]. The rectal defect should be clearly exposed, and the rectal wall should be closed in two layers (inner mucosa and outer seromuscular layer) using 2-0 or 3-0 polyglactin sutures [5,12]. After the defect repair, the integrity of the rectum should be checked finally with retrograde rectal air insufflation, as previously described [5,15]. In the literature, it has been observed that the majority of cases of RI detected intraoperatively and repaired primarily healed without the need for a colostomy. It is important to note that the quality of rectal repair is crucial for primary healing [2]. In our study, rectal defect repair in cases where RI was detected was performed after prostate removal and before vesicourethral anastomosis. The decision to perform primary repair was made in collaboration with an experienced general surgeon, and assistance was obtained from the general surgeon in closing the rectal defect. The defect was closed in two layers, muscular and serosal, using primary 2/0 polyglactin absorbable sutures. After the repair, rectal integrity was checked using an air test. All cases healed without the need for a colostomy.

Regarding postoperative management, oral fluids can be given on the day following surgery, and the diet can be initiated after the patient passes gas [12]. The indication for a low-fiber diet remains controversial. Broad-spectrum antibiotic therapy (penicillin or cefuroxime + metronidazole) is strongly recommended. Furthermore, a longer catheterization period should definitely be considered (>2 weeks), and catheter removal should be planned only after a cystogram with no pathological findings is observed [16]. However, in 10% of patients with RI detected intraoperatively, a RUF develops in the postoperative period [17]. In cases of RI occurring during both operations, we administered cefuroxime and metronidazole antibiotic therapy in the postoperative period. For patients without active complaints and no pathological findings during abdominal examinations, oral fluids were given on the third postoperative day. The catheters of patients who underwent RP were removed on the 12th postoperative day after cystogram control. During long-term follow-up, no RUF developed in these patients.

In general, RI diagnosed after surgery leads to significantly worse outcomes compared to those detected and managed intraoperatively [1]. When diagnosed late, patients undergo more surgical procedures and have longer hospital stays. Undetected RI can lead to serious complications such as abscess formation, peritonitis, septic sequelae, and even death [6]. Failure to recognize and promptly treat bowel injury during RP can lead to a mortality rate as high as 3% [2]. Intraoperative identification and repair of RI during RP is the most critical step to significantly reduce the risk of serious postoperative complications and subsequent RUF formation [6]. In our study, RI cases were identified and managed during the intraoperative period.

Our study has several limitations. Due to its retrospective design and the nature of the available database, we were unable to access certain clinical data, such as the BMI of all patients or a history of prior abdominal surgeries. Another limitation of our study is the relatively small number of patients with RI included. The small number of RI cases limits statistical power for identifying risk factors. The results may not be generalizable to centers with lower surgical volume or less experience, since all procedures were performed by a single high-volume surgeon. Data on RI occurring during RC are scarce in the literature, and our study is among the few contributing to this field. In cases of RI during RC, primary repair can be performed without avoiding the presence of two bowel anastomoses.

One of the most critical aspects of oncologic survival is completing the surgical procedure without leaving behind any tumoral tissue. It is well known that positive surgical margins are among the most significant factors negatively affecting oncologic outcomes. Therefore, in the preoperative period, patients must be adequately informed, and if necessary, rectal incision should not be avoided in order to achieve negative surgical margins.

## 5. Conclusions

Intraoperative detection of RI during pelvic urologic cancer surgery and high-quality primary repair of the defect play a critical role in preventing RI-related morbidity and mortality. Preoperative mechanical bowel preparation may improve visibility of the defect, thus facilitating repair. With intraoperative high-quality primary repair, RI can heal without the need for colostomy and without complications.

## Figures and Tables

**Table 1:** Demographic/clinical characteristics of the RP and RC cases. Radical Prostatectomy (*n* = 494).

	RI + (*n* = 7)	RI − (*n* = 487)	*p* Value
Age, years (mean ± SD)	63 (±5.9)	63.2 (±6.1)	0.949
Pre-op PSA, ng/mL (mean ± SD)	21.1 (±18.5)	13.9 (±21.2)	0.88
Number of patients with pre-op PSA > 20 ng/mL	2 (%28.5)	76 (%15.6)	0.305
RP Gleason Score (ISUP Grade)			0.102
ISUP Grade 1, 2	3 (%42.9)	352 (%97.3)	
ISUP Grade 3, 4, 5	4 (%57.1)	135 (%2.7)	
Number of patients with metastatic lymph nodes	2 (%28.5)	155 (%31.8)	0.953
RP Pathologic T Stage			0.467
Local (pT2a,b,c)	2 (%28.5)	220 (%45.1)	
Locally advanced (pT3a,b, pT4)	5 (%71.4)	267 (%54.8)	
**Radical Cystectomy (*n* = 214)**			
	**RI + (*n* = 8)**	**RI − (*n* = 206)**	***p *** **value**
Age, years (mean ± SD)	65.7 (±8.5)	64.4 (±8.5)	0.539
Gender, *n*			0.679
Male	8 (%100)	174 (%84.4)	
Female	0 (%0)	32 (%15.5)	
Initial TUR-BT Stage, *n*			0.956
Non-muscle invasive	1 (12.5)	25 (%12.2)	
Muscle invasive	7 (%87.5)	181 (%87.8)	
Variant Histology in Initial TURBT, *n*			0.982
Absent	4 (%50)	107 (%52)	
Present	4 (%50)	99 (%48)	
Neoadjuvant Chemotherapy, *n*			0.601
Not received	8(%100)	178 (%86.5)	
Received	0 (%0)	28 (%13.5)	
Number of patients with metastatic lymph nodes	2 (%25)	78 (%37.8)	0.713
Co-existing Prostate Cancer			0.483
Absent	6 (%75)	30 (%66.1)	
Present	2 (%25)	70 (%33.9)	
RC Pathologic T Stage, *n*			0.493
Local (pT2a,b,c)	5 (%62.5)	100 (%48.5)	
Locally advanced (pT3a,b pT4)	3 (%37.5)	106 (%51.4)	

**Table 2:** RP patients with rectal injury.

No	1	2	3	4	5	6	7
Age	53	69	66	59	61	70	63
BMI	27	26	30	28	25	24	27
DRE	Diffuse hardness	Left basal induration	Right basal nodule	Basal hardness	Right basal hardness	Left apex hardness	Right basal hardness
**PSA(ng/mL)**	56.4	11.8	8.3	17	10.9	6.8	37
Duration between biopsy and surgery (in months)	8	4	3	4	11	1	1
Gleason Score	4 + 5 = 9	3 + 4 = 7	4 + 5 = 9	3 + 3 = 6	3 + 3 = 6	3 + 3 = 6	4 + 3 = 7
EAU Risk Classification	High	Intermediate	High	Intermediate	Intermediate	Low	High
Surgical Margin	+	-	+	-	+	-	+
Pathological stage	T3b	T2c	T4	T3a	T3a	T2a	T3b
Metastatic lymph node	+	-	+	-	-	-	-
Prostate volume (cc)	52	63	68	116	46	54	76
Injury size (cm)	3	1	3	1	2	2	1
The stage of the injury	Basal dissection	Apical dissection	Basal dissection	Basal dissection	Basal dissection	Basal dissection	Apical dissection
Operation time (min)	195	180	200	190	195	200	185
Hospital stay duration (days)	10	12	12	15	15	12	8
Follow-up duration (months)	21	99	33	66	48	158	148

**Table 3:** RC patients with rectal injury.

No	1	2	3	4	5	6	7	8
Age	68	73	51	69	57	61	72	75
BMI	20	23	24	22	27	25	29	28
TUR-BT pathology	T2 High CIS	T2 High CIS	T2 High Cıs + Squamous	T2 High CIS	T2 High CIS	T2 High Cıs + Squamous	T2 High CIS + Villoglandular	T1 Low
Pathological stage	T0 CIS	T3a Villoglandular	T4a Squamous	T2b	T4a	T2a Squamous	T0	T0
Surgical Margin	-	-	-	-	-	-	-	-
Metastatic lymph node	+	-	-	-	+	-	-	-
Injury size (cm)	2	2	1	3	3	1	2	2
The stage of the injury	Basal dissection	Apical dissection	Basal dissection	Basal dissection	Basal dissection	Basal dissection	Apical dissection	Basal dissection
Operation time (min)	275	290	260	290	310	260	250	230
Hospital stay duration (days)	26	24	28	20	26	21	16	19
Follow-up duration (months)	39	5	53	89	79	64	61	54

## Data Availability

The data presented in this study are available on request from the corresponding author.

## Abbreviations

The following abbreviations are used in this manuscript:

RI	Rectal injuries
RP	Radical prostatectomy
RC	Radical cystectomy
PUOS	Pelvic uro-oncological surgery
RUF	Rectourethral fistula
TUR-BT	Transurethral resection of bladder tumor
